# MemScreen: A smartphone application for detection of mild cognitive impairment: A validation study: Smartphone App for MCI Detection

**DOI:** 10.1016/j.tjpad.2025.100077

**Published:** 2025-01-28

**Authors:** Julien Dumurgier, Claire Paquet, Jacques Hugon, Vincent Planche, Sinead Gaubert, Stéphane Epelbaum, Stéphanie Bombois, Marc Teichmann, Richard Levy, Estelle Baudouin, Agathe Vrillon, Claire Hourrègue, Emmanuel Cognat, Séverine Sabia, Archana Singh-Manoux

**Affiliations:** a Université Paris-Cité, Cognitive Neurology Center, GHU APHP Nord Lariboisière Fernand-Widal Hospital, 200 rue du Faubourg Saint Denis, 75010 Paris, France; b Université Paris Cité, INSERM U1153, Centre of Research in Epidemiology and Statistics, Team Epidemiology of Ageing and Neurodegenerative Diseases, 10 avenue de Verdun, 75010 Paris, France; c Université Paris Cité, INSERM U1144, Therapeutic Optimization in Neuropsychopharmacology, 2 rue Ambroise Paré, 7510 Paris, France; d Institut des Maladies Neurodégénératives, Bordeaux University, CNRS, UMR 5293, 146 Rue Léo Saignat, 33000 Bordeaux, Bordeaux, France; e Sorbonne Université, Institute of Memory and Alzheimer's Disease, Groupe Hospitalier Pitié-Salpêtrière, AP-HP, 47 boulevard de l'Hôpital, 75010 Paris, France; f Groupement Hospitalier Portes de Provence, route de Sauzet. 26200 Montélimar, France; g Faculty of Brain Sciences, University College London, 38-50 Bidborough Street, WC1H 9BT London, UK

**Keywords:** Smartphone application, Cognitive screening, Mild cognitive impairment, MemScreen, Validation study, Digital health tool

## Abstract

**Background and objectives:**

Primary care is often the first point of contact for patients with cognitive complaints, making initial cognitive screening an essential step to avoid delays in diagnosing Alzheimer's disease (AD) at an early stage. We developed MemScreen, a self-administered smartphone application that assesses overall cognition and verbal memory, and evaluated its ability to detect mild cognitive impairment (MCI) in both general and clinical populations.

**Methods:**

We conducted two validation cohort studies: (1) UK-based Whitehall II cohort study (13th wave, 2018–2022) involving a general population (MCI defined by poor performance on a global cognitive score), and (2) five French memory clinics involving patients without dementia (amnestic MCI defined by the Free and Cued Selective Reminding Test). MemScreen, MMSE, and TMT-A effectiveness was assessed using Area Under the Curve (AUC) values from unadjusted and adjusted logistic regression models.

**Results:**

In Whitehall II (*n* = 2118, mean age 75.9 years, 23.9 % women, 14.5 % MCI), median MemScreen completion time was 4 min 18 s. MemScreen had the highest AUC (0.87; 95 % CI: 0.82–0.89) for distinguishing MCI, outperforming MMSE (AUC = 0.79; 0.76–0.82; *p* = 0.018) and TMT-A (AUC = 0.77; 0.74–0.80; *p* = 0.023). MemScreen sensitivity and specificity were 78.6 % and 78.7 %, respectively. In memory clinics (*n* = 303, mean age 70.5 years, 53 % women, 46.9 % amnestic MCI), median completion time was 5 min 17 s. MemScreen showed superior performance (AUC = 0.87; 0.83–0.91) compared to MMSE (AUC = 0.72; 0.67–0.78; *p* < 0.001) and TMT-A (AUC = 0.63; 0.56–0.69; *p* < 0.001), with 93.0 % sensitivity and 54.0 % specificity for amnestic MCI.

**Discussion:**

MemScreen outperformed traditional tests in identifying MCI in both general and clinical populations. Its self-administration and short completion time suggest potential as an effective screening tool to optimize memory clinic referrals for AD diagnosis and treatment.

## Highlights

• MemScreen is a smartphone-based, self-administered tool for detecting mild cognitive impairment (MCI).
• It outperformed traditional cognitive tests (MMSE, TMT-A) in accurately identifying MCI.
• MemScreen was validated across both general population and memory clinic cohorts.
• Its brief administration time supports its use as an efficient primary screening tool.

## 1 Introduction

Most cases of Alzheimer's disease (AD) in population settings continue to be diagnosed at the dementia stage that is characterized by significant cognitive decline and impairment in daily function and autonomy [1]. The elaboration of the concept of mild cognitive impairment (MCI) [2], along with the advances in identification of specific biomarkers of AD [3], allows the possibility of diagnosing AD at an early stage [4,5]. Patients or their families notice cognitive deterioration, and objective neuropsychological tests reveal poor performance, yet daily functioning remains largely unaffected at the MCI stage [6].

Recent results from immunotherapies that target the beta-amyloid peptide [7,8] have demonstrated clinical efficacy in treating Alzheimer's disease at an early stage. These findings highlight the importance of identifying patients at the early stage of AD, as these patients could benefit the most from these new treatments. Early-stage diagnosis of AD is complex, usually beginning with initial referral from primary care [9], followed by a comprehensive diagnostic approach incorporating biomarkers in specialized memory clinics [4]. In real-life settings, the diagnosis of AD is often at a late stage of disease; the mean score on the Mini Mental State Examination (MMSE) in large-scale studies is reported to be around 19 (out of 30) at diagnosis [10,11]. The implication is that the disease has already progressed to a moderate stage of dementia. Consequently, only a small fraction of patients seen in memory clinics would be potential candidates for anti-amyloid immunotherapy because these therapies most of the time apply to patients with an MMSE > 22 according to current recommendations [[12], [13], [14]]. Patients who could benefit from new treatments are currently not being identified and referred early enough in the disease process.

A 2021 survey of 801 US primary care physicians by the Alzheimer's Association revealed that while 90 % recognized the importance of diagnosing MCI, 77 % found making such a diagnosis particularly challenging [15]. Tests of cognitive function such as the MMSE [16], the General Practitioner assessment of Cognition (GP-Cog) [17], or the Six Item Cognitive Impairment Test (6-CIT) [18] could be used on patients presenting with cognitive concerns in primary care. However, these tests have limitations, including inter-examiner variability and the requirement for specific training to administer them. These tests are administered infrequently in primary care [19], raising questions about their ease of use or the time taken to administer them.

To bridge this gap, we developed MemScreen, a smartphone app designed for physicians to screen for MCI in primary care settings, providing both an overall cognitive assessment and a specific evaluation of verbal memory. The aim of the present study was to test the validity of MemScreen in two types of study populations with complementary aims. The first was Whitehall II, a general population cohort study where performance on the MemScreen was benchmarked against poor performance on a standard cognitive test battery. The second was a multi-centre study on memory clinic patients without dementia where the effectiveness of MemScreen to identify amnestic MCI was examined against a gold-standard Free and Cued Selective Reminding Test (FCSRT) [20], an objective measure of episodic verbal memory impairment, known to be strongly associated with MCI due to AD [21].

## 2 Methods

### 2.1 Study population

The Whitehall II Study is an ongoing cohort study involving individuals originally employed by the British Civil Service. Full details of this study have been reported previously [22]. A total of 10,308 individuals aged 35–55 years (67 % male) were recruited to the study between 1985 and 1988 (response rate 73 %). The study involves clinical examinations every 4 to 5 years. The data used in our study were derived from the 13th wave of follow-up (2018–2022) of the study, during which MemScreen was incorporated into the standard neuropsychological test battery for participants seen at the clinical examination in London, who were determined to be without dementia diagnosis (from electronic health records) and without limitations on the instrumental activities of daily living (IADL).

Memory center patients were drawn from five memory clinics in France: La Salpêtrière and Lariboisière hospitals in Paris, and hospitals in Bordeaux, Gonesse, and Bézier. A total of 303 non-demented patients were administered the MemScreen as part of their comprehensive neuropsychological assessment for investigating a cognitive disorder or complaint, between June 2019 and June 2020. These patients were required to have a Mini-Mental State Examination (MMSE) score of 23/30 or higher.

### 2.2 Mild cognitive impairment

In the Whitehall II Study, we calculated a global cognitive score using standardized scores on three tests: the Alice Heim 4-I test [23], which consists of 65 verbal and mathematical reasoning items of increasing difficulty to be completed within 10 min; one-minute phonemic (letter S) fluency task; and a verbal episodic memory test involving immediate recall (participants were presented with a list of 20 one- or two-syllable words at two-second intervals and then asked to recall as many words as possible in writing within two minutes). The sum of these three standardized scores was re-standardized to derive the global cognitive score (mean=0, and standard deviation (SD)=1). Participants with a composite score of −1 SD or lower were classified as having MCI [21]. The test battery also included the MMSE and Trail Making Test (TMT)-A but they were not included in the global cognitive score, as we intended to compare performance of these tests with MemScreen.

For the validation study on memory clinic patients, our goal was to examine whether MemScreen could identify patients with amnestic MCI, as it is indicative of AD etiology [21] and is a common inclusion criterion in therapeutic clinical trials for early-stage AD. All patients included in this study had undergone a comprehensive neuropsychological evaluation in the participating memory centers, had an MMSE score of 23 or higher, and had been assessed for verbal episodic memory using the Free and Cued Selective Reminding Test (FCSRT). We used previously published cut-off values, considering a total free recall of 17/48 or less or a total recall of 40/48 or less to define patients with amnestic MCI of hippocampal type [24]. In the memory clinic cohort, we also undertook a comparison of MemScreen with the MMSE, TMT-A, as well as with the Frontal Assessment Battery (FAB) [25].

### 2.3 MemScreen

MemScreen is a smartphone application that we developed for Android and Apple devices, freely available and downloadable from their respective stores. It is designed for use by a healthcare professional, including primary care physicians or nurses, to evaluate minor cognitive disorders. The test is self-administered by the patient under the supervision of a healthcare provider, who initiates the test and ensures that each section of the test is completed. This serves as an alternative or complement to traditional paper-and-pencil screening tools. The app allows patients to include their education level and age, divided into four categories each. The test consists of three parts:

1. Learning a list of 12 words in sets of four, with semantic encoding control.
2. A 22-point test assessing temporal orientation (5 points), addition (5 points), subtraction (5 points), clock reading (4 points), and intruder identification (3 points).
3. A delayed recall test (12 points) involving recognition of the initially learned 12 words with 36 semantic distractors.

**Fig. 1:**
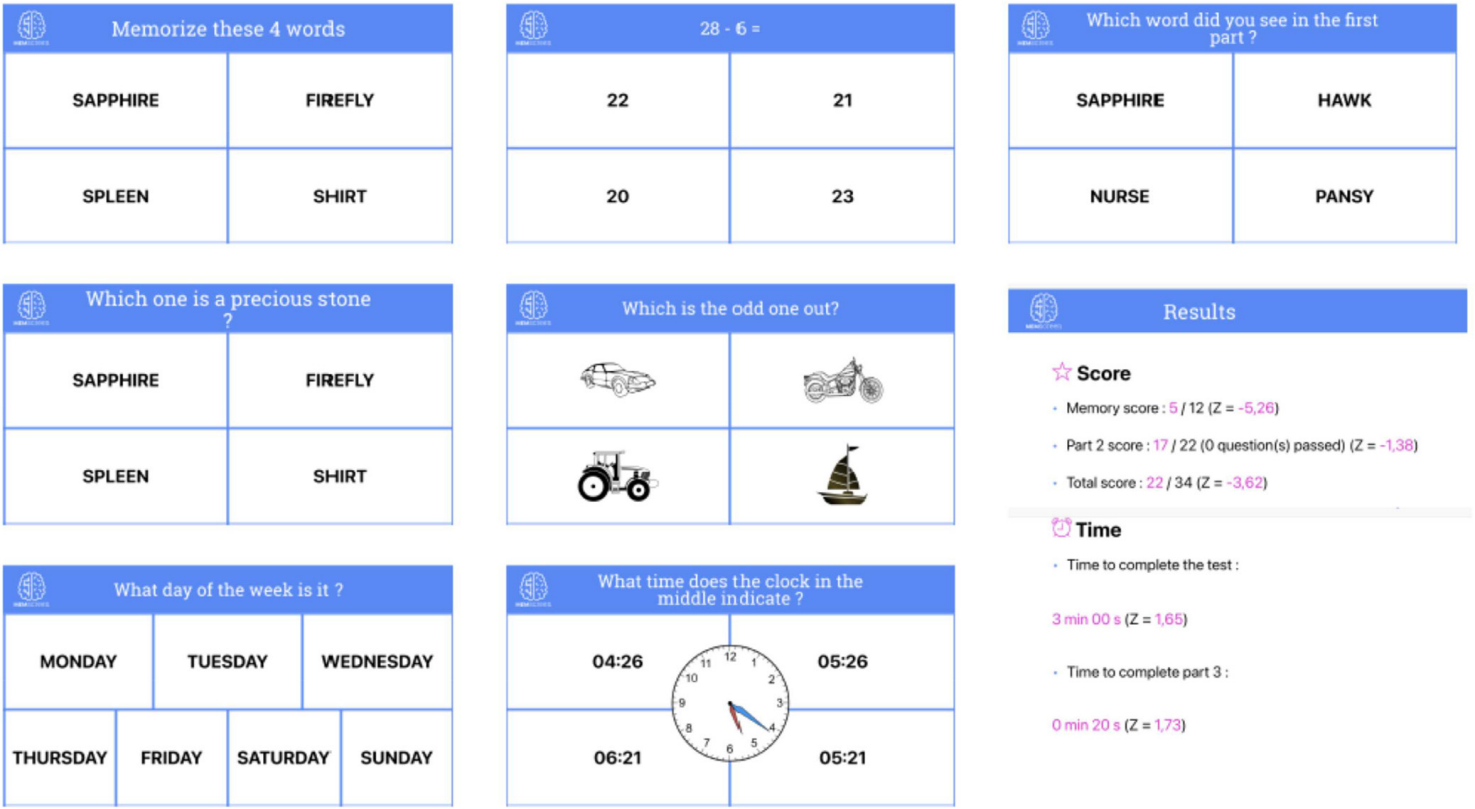
Screenshots of the MemScreen test.

The test yields a total score of 34 points, which contains a memory score of 12 points. It also records the time taken for the test and for the delayed recall component. Fig. 1 shows screenshots of the app, available in English and in French. A test version of the app has been made available with the username: test@memscreen.org, and password: 123memscreen.

### 2.4 Covariates

In both cohorts, age, sex, and educational level were used as covariates. Education was categorized into three groups in both study populations as lower secondary school or less, higher secondary school, and university or higher degree.

### 2.5 Statistical analysis

The validation strategy for MemScreen was undertaken separately in the Whitehall study and the multi-centre cohort of memory clinic patients.

#### 2.5.1 Validation in Whitehall II (general population-based study)

We examined the ability of MemScreen (combination of total score and test completion time in seconds), the MMSE and TMT-A to identify participants with MCI as defined by a global cognitive score lower than −1 SD. The Area Under the Curve (AUC) and its 95 % confidence interval were calculated for predicting MCI (yes/no) using logistic regression, results were presented graphically as ROC curves. The analyses were unadjusted and then adjusted for age, sex, and education. We compared the AUCs associated with the different tests (MMSE, TMT-A, MemScreen) using the ROCCOMP command in STATA with the AUC derived from the MMSE scores as the reference. We also examined the Akaike Information Criterion (AIC) values for each model, where the lowest AIC indicates the best fitting model. Post-estimation from logistic regression models were obtained using the "predict" option in Stata's "logit" function, and the cut-off value was defined as the threshold that maximized the Youden Index (an index used for setting optimal thresholds: the sum of sensitivity and specificity minus one).

#### 2.5.2 Validation in memory clinic patients

In the multi-centre cohort of non-demented memory clinic patients, we examined the ability of MemScreen, MMSE, TMT-A, and FAB to discriminate between patients with amnestic (abnormal FCSRT) MCI. We then used the cut-off identified in the Whitehall II study population to examine MemScreen's sensitivity and specificity in distinguishing amnestic MCI patients in the memory clinic cohort.

Two-tailed values of *p* < 0.05 were considered statistically significant, statistical analyses were performed using Stata 15 (StataCorp LP, College Station, TX).

## 3 Results

### 3.1 MemScreen analyses in the Whitehall II study

A total of 2118 participants were administered MemScreen at the clinical examination. Their mean (SD) age was 75.9 (4.6) years, with 23.9 % being female, and 1961 (92.6 %) identified as white. The mean MMSE score was 28.1 (1.8) and 308 (14.5 %) of these participants were classified as having MCI, in that their global cognitive score was lower than −1 SD. Those classified as MCI compared to all others were older, more often female, had lower educational levels, and had poorer performance across all cognitive tests (Table 1, all *p* < 0.001). The mean time to complete MemScreen was 338.1 s in those classified as MCI and 260.6 s in all others (*p* < 0.001). The median completion time for MemScreen in the overall population was 258 s, with an interquartile range of 234 to 292 s.

**Table 1:** Characteristics of participants of the Whitehall II study included in the analyses.

		Total	Mild cognitive impairment*		
	N	N = 2118	No (*n* = 1810)	Yes (*n* = 308)	p-value
**Socio-demographic characteristics**					
Age, year, mean (SD)	2118	75.9 (4.6)	75.4 (4.4)	78.6 (5.0)	<0.001
Women, n (%)	2118	506 (23.9)	395 (21.8)	111 (36.0)	<0.001
Education, n (%)	2118				
Lower secondary school or less		759 (35.8)	587 (32.4)	172 (55.8)	
Higher secondary school		589 (27.8)	522 (28.8)	67 (21.8)	
University and higher degree		770 (36.4)	701 (38.7)	69 (22.4)	<0.001
**Cognitive scores,** mean (SD)					
Verbal memory (range 0 to 20)	2098	3.9 (2.1)	4.3 (2.0)	1.6 (1.2)	<0.001
Verbal S fluency (range 0 to 35)	2097	14.7 (4.3)	15.6 (3.7)	9.5 (3.4)	<0.001
Alice Heim 4-I (range 0 to 65)	2103	43.8 (10.9)	46.7 (8.7)	27.5 (8.4)	<0.001
MMSE score (range 0 to 30)	2118	28.1 (1.8)	28.5 (1.4)	26.3 (2.6)	<0.001
TMT-A, time in seconds	2115	37.8 (15.2)	35.7 (13.2)	49.9 (19.7)	<0.001
**Memscreen,** mean (SD)					
Total score (range 0 to 34)	2118	31.7 (2.3)	32.0 (1.9)	29.8 (3.0)	<0.001
Total time, time in seconds	2118	271.9 (62.9)	260.6 (47.9)	338.1 (93.2)	<0.001

⁎ Determined based on the global cognitive score, scores ≤1 SD. MMSE: Mini Mental State Examination, TMT-A: Trail Making Test-A.

**Fig. 2:**
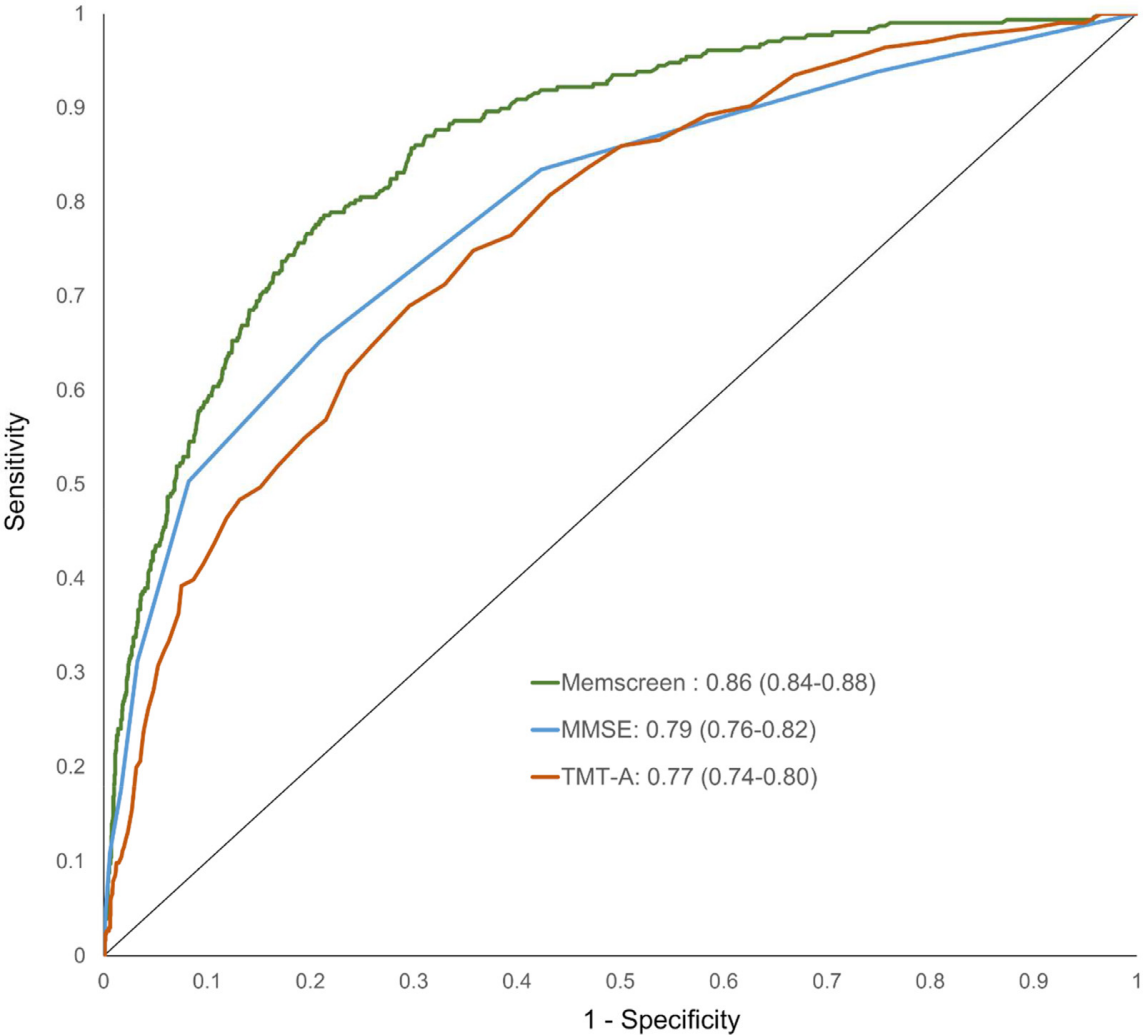
Ability of MemScreen, MMSE, and TMT-A tests to discriminate between MCI and non-MCI cases in the Whitehall II study.

The Receiver Operating Characteristic (ROC) curves, demonstrating the ability of MemScreen, MMSE, and TMT-A to discriminate MCI cases and non-cases in the Whitehall II study are shown in Fig. 2. The MemScreen total score (out of 34) combined with time to complete this test yielded the highest Area Under the Curve (AUC), a value of 0.86 (95 % CI: 0.84 to 0.88). The MMSE score in these analyses had an AUC of 0.79 (0.76 to 0.82), and the TMT-A 0.77 (0.74 to 0.80).

Table 2 presents the AUC values derived from univariate and multivariate logistic regression models. Age, sex, and education together had an AUC of 0.73 (0.70 to 0.76) to distinguish MCI from non-MCI individuals. In the analyses of screening tests adjusted for age, sex, and education, MemScreen had the highest AUC (0.87; 0.82 to 0.89) and TMT-A the lowest (0.81; 0.78 to 0.83). In these analyses with MMSE as the reference, the AUC for TMT-A was significantly lower (*p* = 0.023) and that for MemScreen higher (*p* = 0.018). Of the three tests, MemScreen had the highest AUC and unlike the MMSE and TMT-A, incorporating age, sex, and education did not improve model performance. MemScreen also had the lowest AIC values, indicating better model fit.

**Table 2:** The ability of MemScreen, MMSE, TMT-A to discriminate between participants with and without MCI in the Whitehall II study. AUC (95 % CI) are from logistic regression models.

	AUC (95 % CI)	P-value	AIC
Age, sex, education	0.73 (0.70, 0.76)	_	1570
			
MMSE	0.79 (0.76, 0.82)	Ref.	1429
TMT-A	0.77 (0.74, 0.80)	0.24	1559
MemScreen	0.86 (0.84, 0.88)	<0.001	1319
			
MMSE, age, sex, education	0.83 (0.81, 0.86)	Ref.	1332
TMT-A, age, sex, education	0.81 (0.78, 0.83)	0.023	1441
MemScreen, age, sex, education	0.87 (0.82, 0.89)	0.018	1272

The coefficients from the logistic regression model with MCI as the outcome and MemScreen total score (SCORE) and completion time (TIME) as predictors are as follows:

$$Logit=\frac{\text{exp}\left(1.605+\;0.017\;\times \;\text{TIME}-0.267\;\times \;\text{SCORE}\right)}{1+\;\text{exp}\left(1.605+\;0.017\;\times \;\text{TIME}-0.267\;\times \;\text{SCORE}\right)}$$

A logit value of 0.13 was associated with the highest Youden Index, with a sensitivity of 78.6 % and a specificity of 78.7 %. By comparison, an MMSE score of 27, which maximized the Youden Index, was associated with a sensitivity of 79.0 % and a specificity of 65.3 %.

#### 3.1.1 Validation of MemScreen in memory clinic patients

Data on MemsScreen and other neuropsychological tests were available on 303 patients without dementia, drawn from five memory clinics. The characteristics of this patient cohort are shown in Table 3. The mean age (SD) was 70.5 (9.7) years, 53 % were female, and the mean MMSE score was 26.5 (2.5). Of the 303 patients, 142 (46.9 %) were identified as having amnestic MCI, defined by abnormal performance on either the free recall or delayed recall of the FCSRT. Patients with amnestic MCI were older, more often male, had a lower level of education, and had poorer scores across all neuropsychological tests. The mean time (SD) to complete MemScreen was 417.6 s in those classified as having amnestic MCI and 317.5 s in others (*p* < 0.001). The median completion time in the overall population was 335 s, with an interquartile range of 275 to 418 s.

**Table 3:** Characteristics of patients from the multi-centre memory clinics.

	Total	Amnestic MCI		
	N = 303	No (*n* = 161)	Yes (*n* = 142)	p-value
**Socio-demographic characteristics**				
Age in years, mean (SD)	70.5 (9.7)	67.2 (9.9)	74.2 (8.1)	<0.001
Women, n (%)	161 (53.1)	102 (63.4)	59 (41.6)	<0.001
Education, n (%)				
Lower secondary school or less	50 (16.6)	17 (10.6)	33 (23.2)	
Higher secondary school	121 (40.1)	67 (41.9)	54 (38.0)	
University and higher degree	131 (43.3)	76 (47.5)	55 (38.7)	0.01
**Cognitive scores,** mean (SD)				
MMSE (range 0 to 30)	26.5 (2.5)	27.4 (2.1)	25.4 (2.6)	<0.001
Frontal Assessment Battery (range 0 to 18)	15.2 (2.4)	15.9 (2.2)	14.3 (2.4)	<0.001
TMT-A, time in seconds	54.1 (25.6)	48.8 (20.2)	60.7 (29.8)	<0.001
Free and Cued Selective Reminding test				
Immediate free recall (range 0 to 48)	20.7 (10.4)	28.7 (5.5)	11.6 (6.5)	<0.001
Immediate total recall (range 0 to 48)	38.2 (11.2)	45.6 (2.3)	29.7 (11.3)	<0.001
**MemScreen,** mean (SD)				
Total score (range 0 to 34)	29.1 (3.4)	31.0 (2.5)	26.9 (3.0)	<0.001
Total time, time in seconds	364.4 (122.5)	317.5 (97.8)	417.6 (126.3)	<0.001

Amnestic MCI based on Free and Cued Selective Reminding Test performances.

MMSE: Mini Mental State Examination, TMT: Trail Making Test.

**Fig. 3:**
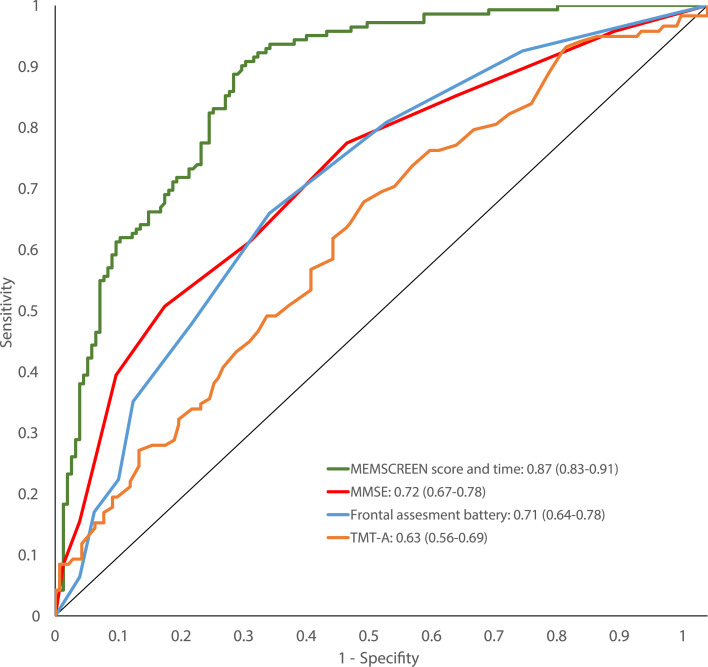
The ability of MemScreen, MMSE, Frontal Assessment battery, and TMT-A to discriminate amnestic from non-amnestic MCI patients in a multi-centre memory clinics data.

Fig. 3 shows ROC curves illustrating the ability of MemScreen, MMSE, Frontal Assessment battery, and TMT-A tests to discriminate amnestic MCI patients. MemScreen had the highest AUC (95 %CI): 0.87 (0.83 to 0.91). For comparison, the AUCs associated with the MMSE, FAB, and TMT-A were 0.72 (0.67 to 0.78), 0.71 (0.64 to 0.78), and 0.63 (0.56 to 0.69), respectively. Comparative analyses using MMSE as the reference revealed that the AUC for TMT-A was significantly lower (*p* = 0.013), while the AUC for MemScreen was significantly higher (*p* < 0.001). No significant difference was found in the AUC for the Frontal Assessment Battery compared to MMSE (*p* = 0.40).

The thresholds for MemScreen, identified in the Whitehall II study, were applied to the patient cohort and resulted in a sensitivity of 93.0 % and a specificity of 54.0 % for identifying individuals with amnestic MCI.

## 4 Discussion

We developed MemScreen as a clinical screening tool to identify individuals with MCI in primary care. In over 2000 general population participants from the Whitehall II study we found MemScreen to have a robust ability to discriminate between individuals with MCI, surpassing traditional tests such as the MMSE. Subsequently, we validated MemScreen's utility in a clinical setting based on non-demented patients from memory clinics, showing its effectiveness in detecting patients with amnestic syndrome of hippocampal type, a common phenotype of MCI due to AD.

The use of digital tools like MemScreen offers multiple advantages: self-administration by patients removes the need for physicians to learn test instructions, improving inter-examiner reproducibility; the test is quick, with a median completion time under 4 min and 30 s in the general population and just over 6 min in a patient population. Additionally, the scoring process is automated with results on the total score and the time taken to complete the test. It is important to note that MCI is not specific to a single etiology and can result from various underlying causes. In primary care settings, a positive MemScreen result ought to initiate a broader diagnostic process that could include biomarker analyses in cases where Alzheimer's disease is suspected.

The near-universal adoption of smartphones among physicians [26] has paved the way for innovative digital approaches to cognitive evaluation [27] or cognitive training [28]. This transformation in medical practice is underscored by the recent development and deployment of a wide array of applications for cognitive assessment [29]. Reviews of such tools have cataloged more than thirty such tools [30,31]. These applications can be broadly categorized into three groups: 1) mobile adaptations of traditional neuropsychological tests, such as eMOCA [32] or eSAGE [33], 2) novel cognitive assessments specifically designed for mobile platforms, like the Toronto Cognitive Assessment test [34] or the Santé-Cerveau digital tool [35], and 3) innovative approaches that utilize new data streams for cognitive assessment, such as those incorporating GPS location tracking [36], gaming [37], or voice analysis [38]. Compared to existing applications, MemScreen's advantages include ease of use by physicians, brief duration, and minimal physician involvement.

The sensitivity and specificity of MemScreen varied depending on the population and how MCI was ascertained. In the Whitehall II study, sensitivity and specificity were approximately 78 % for identifying global MCI, as defined by a composite cognitive score. While not perfect, this performance surpassed that for the widely used MMSE, highlighting MemScreen's utility as a screening tool. In the memory clinic cohort, sensitivity was high (93 %), with lower specificity (54 %), reflecting the specific nature of this population referred for specialized cognitive assessment by primary care physicians. Within this context, high sensitivity is particularly important as it ensures a low rate of false negatives. A normal MemScreen result suggests that amnestic MCI is unlikely, offering clinicians valuable information. Our results show MemScreen to be a good initial screening tool that can help clinicians decide whether to pursue a comprehensive evaluation of cognitive status of an individual. Abnormal results on the MemScreen should prompt further investigations to confirm or rule out the presence of cognitive impairment.

Furthermore, analyses presented in the present study show it to be valid both in the general and patient populations. The cut-point derived from the general population was found to have a good sensitivity in both the general population and patient sample, while the specificity was reasonable, making it a potential valuable screening tool to refer patient identified as potential MCI to specialized centers for further investigations.

One of the strengths of our study is that the validation of MemScreen was undertaken both in a large general population cohort to identify individuals with MCI, and in a clinical population of memory clinic patients to identify those with amnestic MCI. Additionally, we were able to compare its performance to the MMSE, a widely used instrument in various settings. The Whitehall II study includes a wide socioeconomic spectrum by design but all participants were in employment at baseline and the study may not fully represent the diversity of a typical primary care population. Participants from this study may differ from the general population on a range of risk and protective factors. Further research is required to assessing the real-world integration of MemScreen by general practitioners in routine primary care practice, and validate it in diverse populations, including those from different cultural and linguistic contexts, to ensure its utility and adaptability globally. A further limitation of the present study is the absence of qualitative data on experiences of healthcare providers, study participants or patients using MemScreen. Qualitative evaluations to assess usability, acceptability, and user satisfaction would be important for optimizing the application for real-world use in primary care. In addition it is worth noting that the inclusion criteria for patients in the memory clinic study was a MMSE score of 23/30 or higher, implying that some of these patients might have had mild dementia rather than MCI. This does not take away from the overall objective of identifying patients at an early stage of cognitive dysfunction, particularly those who could benefit from targeted interventions in the context of emerging treatments for Alzheimer's disease.

In conclusion, MemScreen is a simple, rapid screening tool to identify individuals with MCI and is fit for use both in the general population and in memory clinic patients. We have shown in this study that this simple tool outperforms the traditional MMSE test, and its quick administration makes it well-suited for cognitive evaluation in primary care. Findings from this study address our overarching aim to develop an easy-to-use, accurate tool to assist primary care physicians in optimizing patient referrals to memory clinics for more advanced assessments.

## Funding

The development of the MemScreen application and this research were further supported by a grant from Lilly France and the Fondation Vaincre Alzheimer.

## Ethical standards

For the Whitehall II Study, participant written, informed consent and research ethics approvals are renewed at each point of contact. The most recent approval was granted by the National Health Service (NHS) London-Harrow Research Ethics Committee, with the reference number 85/0938. For the memory center patients, the study protocol was approved by the “Comité de Protection des Personnes Sud Est V” Committee (approval number 2018-A02672–53). The study is registered on ClinicalTrials.gov with the identifier NCT03811184. All participants provided written, informed consent.

## CRediT authorship contribution statement

**Julien Dumurgier:** Writing – original draft, Methodology, Investigation, Funding acquisition, Formal analysis, Conceptualization. **Claire Paquet:** Writing – review & editing, Methodology, Conceptualization. **Jacques Hugon:** Writing – review & editing, Funding acquisition, Conceptualization. **Vincent Planche:** Writing – review & editing, Investigation. **Sinead Gaubert:** Writing – review & editing, Investigation, Data curation. **Stéphane Epelbaum:** Writing – review & editing, Investigation, Data curation. **Stéphanie Bombois:** Writing – review & editing, Investigation, Data curation. **Marc Teichmann:** Writing – review & editing, Investigation, Data curation. **Richard Levy:** Writing – review & editing, Investigation, Data curation. **Estelle Baudouin:** Writing – review & editing, Investigation, Data curation. **Agathe Vrillon:** Writing – review & editing, Investigation, Data curation. **Claire Hourrègue:** Writing – review & editing, Investigation, Data curation. **Emmanuel Cognat:** Writing – review & editing, Investigation, Data curation. **Séverine Sabia:** Writing – review & editing, Writing – original draft, Formal analysis. **Archana Singh-Manoux:** Writing – review & editing, Writing – original draft, Investigation, Conceptualization.

## Declaration of competing interest

The authors declare the following financial interests/personal relationships which may be considered as potential competing interests:

JULIEN DUMURGIER reports financial support was provided by Lilly France. JULIEN DUMURGIER reports financial support was provided by Fondation Vaincre Alzheimer. If there are other authors, they declare that they have no known competing financial interests or personal relationships that could have appeared to influence the work reported in this paper.
